# Herpes Zoster and Cardiovascular Disease: Exploring Associations and Preventive Measures through Vaccination

**DOI:** 10.3390/vaccines12030252

**Published:** 2024-02-28

**Authors:** Minako Yamaoka-Tojo, Taiki Tojo

**Affiliations:** 1 Department of Rehabilitation, Kitasato University School of Allied Health Sciences, Sagamihara 252-0373, Japan; 2 Department of Cardiovascular Medicine, Kitasato University Graduate School of Medical Sciences, Sagamihara 252-0373, Japan; 3 Department of Cardiovascular Medicine, Kitasato University School of Medicine, Sagamihara 252-0374, Japan

**Keywords:** vasculopathy, inflammation, stroke, antiviral treatment, vaccines

## Abstract

Herpes zoster, induced by the reactivation of the varicella-zoster virus (VZV), is a unilaterally distributed vesicular rash that can cause multiple complications. VZV not only causes neurological problems, including postherpetic neuralgia and ocular zoster, but also causes inflammatory vasculopathy and increases the incidence of hemorrhagic or ischemic complications. Therefore, understanding the association between the development of herpes zoster and the subsequent occurrence of acute stroke or cardiovascular diseases, including myocardial infarction and heart failure, is of great interest. Conversely, many risk factors are involved in the development of herpes zoster. Recently, it has become clear that aging, insufficient immune function, and diseases related to lifestyle habits (for example, stroke and cardiovascular disease), can trigger the onset of herpes zoster. Preventing the onset of herpes zoster, which substantially reduces quality of life, will lead to lower medical costs for countries and extend healthy life expectancy for general populations. Thus, because herpes zoster is a vaccine-preventable disease, active vaccination is recommended for high-risk groups. This review summarizes the association between herpes zoster and cardiovascular disease and vaccination against herpes zoster as a useful disease management and prevention measure for cardiovascular disease.

## 1. Introduction

Varicella-zoster virus (VZV) causes chickenpox (varicella), usually in childhood, and becomes latent in the neurons of the cranial and spinal ganglia of nearly all individuals after the chickenpox resolves. Herpes zoster, commonly known as shingles, is a viral disease caused by the reactivation of latent VZV residing in the sensory ganglia and dorsal roots after primary infection, especially in older adults and immunocompromised individuals. It primarily manifests as a painful skin rash and blisters occurring in specific dermatomes (areas along nerves), which are called “zoster” herpes. Herpes zoster represents a significant health concern for the general population [1] because postherpetic neuralgia has a negative impact on quality of life [2]. The overall lifetime risk of herpes zoster is 32.2% [3], and postherpetic neuralgia affects 13–25% of patients with herpes zoster [4].

Recently, the global incidence of herpes zoster has increased; however, the cause remains unknown [4,5,6,7]. Furthermore, in Japan’s aging society, there is a high possibility that the incidence of not only herpes zoster but also chickenpox among older people will increase as cell-mediated immunity specific to VZV declines with age [4,5,8,9,10].

Humans are the only natural hosts for VZV, complicating efforts to understand the pathophysiology of VZV-associated vasculopathy [11]. In postmortem analyses, the cerebral arteries have been observed to contain VZV DNA and VZV-specific antigens, supporting the hypothesis that vasculitis is caused by VZV [12]. The incidence of herpes zoster is increasing with age; the incidence was found to be 10.9 cases per 1000 patient-years in people and was significantly higher in women than in men [13]. Several studies have unequivocally demonstrated that the occurrence of herpes zoster rises with advancing age [14].

The preferred site of herpes zoster is the intercostal nerves, with the next most common site being the ophthalmic division of the trigeminal nerve [15]. Additionally, the seventh cranial nerve may be the site of VZV reactivation. In this review, we summarize the association between herpes zoster and cardiovascular disease and the relevant disease management and prevention measures, including vaccination, for cardiovascular disease.

## 2. Risk Factors for Herpes Zoster

The risk factors that promote the reactivation of herpes zoster include a weakened immune system, advanced aging, and stress [16]. Because herpes zoster develops in immunocompromised patients or those with comorbid conditions or chronic diseases, insufficient immunological protection may increase the risk of severe complications [17].

### 2.1. Literature Search Results for Cardiovascular Disease and Herpes Zoster

The keywords “cardiovascular disease”, “herpes zoster”, “heart failure”, and “myocardial infarction” were searched in PubMed. The research time frame for the database was until December 2023. The language was limited to English. The titles and abstracts of all potentially relevant articles were read to determine their relevance. Full articles were also scrutinized if the titles and abstracts were unclear. Reference lists of identified articles were screened to ensure the completeness of the search.

**Figure 1:**
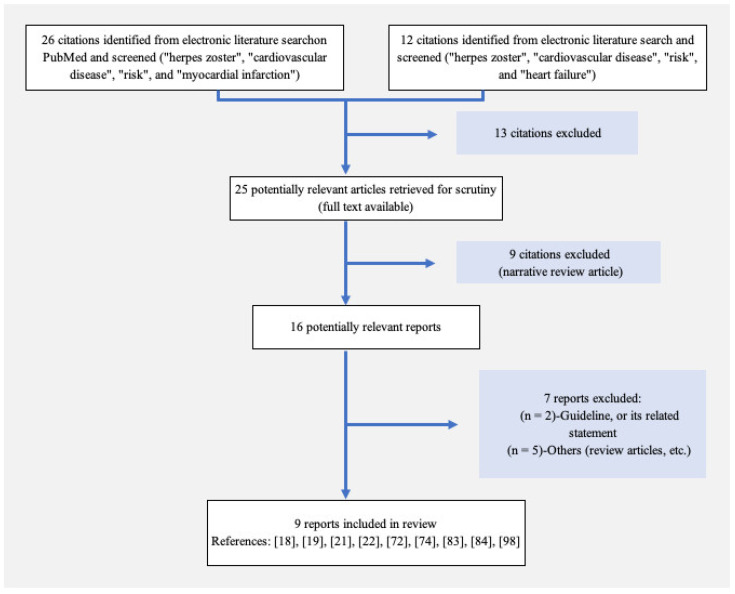
A flowchart depicting the selection process. A total of 25 studies were imported for title and abstract screening after removing duplicates or determining ineligibility. Following a comprehensive full-text review, 16 studies were deemed eligible, and 9 reports were ultimately included in the review.

As depicted in Figure 1, a total of 38 studies underwent title and abstract screening, with 5 studies excluded due to duplication. A total of 2 studies were excluded because they focused on COVID-19 vaccines, and 6 studies were associated with research on the differential diagnoses of chest pain. Subsequently, out of the 13 studies not meeting the eligibility criteria for a full-text review, 7 reports were excluded due to being review articles or guideline-related articles, while 9 reports were included in the review. Figure 1 illustrates a flowchart outlining the selection process.

### 2.2. Cardiovascular Diseases as Risk Factors for Herpes Zoster

In a large cohort study involving 25,209 patients diagnosed with herpes zoster during the study period, dyslipidemia and prior myocardial infarction were significant risk factors, whereas diabetes mellitus, hypertension, prior stroke, and smoking were not [18]. In a Japanese cohort study using the Diagnosis Procedure Combination (DPC) database, age ≥ 75 years, diabetes mellitus, dyslipidemia, hyperuricemia, hypertension, heart failure, and glucocorticoid administration were associated with increased risks of herpes zoster onset in patients hospitalized for cerebrovascular and cardiovascular events [19]. In a Korean nationwide cohort study, comorbidities including hematologic malignancies, hypertension, diabetes mellitus, and chronic lung and liver disease increased the risk of herpes zoster in patients with rheumatic disease [20]. The risk factors for herpes zoster appear to vary across different populations and health conditions. While dyslipidemia and prior myocardial infarction stood out as significant contributors in one study, the Japanese and Korean cohorts highlighted the importance of age, various comorbidities, and medication administration. These nuanced insights emphasize the need for a comprehensive understanding of the diverse factors influencing herpes zoster risk, enabling more tailored preventive strategies in clinical practice.

A population-based study in Taiwan revealed that patients with heart failure, especially men, may have an increased risk of herpes zoster [21]. According to a nationwide population-based case–control study in Korea, patients with an episode of myocardial infarction (hazard ratio [HR] 1.625, 95% confidence interval [CI] 1.144–2.308), ischemic stroke (HR 1.518, 95% CI 1.177–1.957), or heart failure (HR 1.485, 95% CI 1.041–2.117) were at increased risk of hospitalization for herpes zoster [22]. These findings emphasize the complex relationship between cardiovascular events and the susceptibility to herpes zoster. Individuals with heart failure, ischemic heart disease, stroke, or other cardiovascular diseases, as well as those with a history of such conditions, not only face an elevated risk of developing herpes zoster but are also more likely to be hospitalized due to the exacerbation of either herpes zoster or cardiovascular diseases.

### 2.3. Diabetes

Diabetes mellitus was associated with an increased risk of herpes zoster in a multivariate analysis [23,24,25,26]. Patients with poor glycemic control, with a mean HbA1c of 10.7%, had a higher risk of developing herpes zoster (HR 1.44, 95% CI 1.26–1.64) [26]. However, even after experiencing hyperglycemia, the risk of developing shingles has been shown to be low if adequate blood sugar control is achieved through treatment. Therefore, the prompt initiation of hyperglycemic control is essential, and achieving optimal glycemic control early contributes to the prevention of shingles. Proactive measures, including regular monitoring and timely adjustments to treatment plans, may play a pivotal role in reducing the overall burden of herpes zoster in patients with diabetes.

**Table 1:** Risk factors for herpes zoster.

Risks [Reference]	Results (Country; Herpes Zoster Cases)	Subgroups	HR (95% CI)
Cardiovascular diseases [22]	Cardiovascular diseases increase the risk of herpes zoster hospitalization (Korea; 20,311)	Myocardial infarction	1.625 (1.144–2.308)
		Stroke	1.518 (1.177–1.957)
		Heart failure	1.485 (1.041–2.117)
Rheumatoid arthritis [27]	RA increases the risk of herpes zoster (USA and UK; 1611)	USA	1.91 (1.80–2.03)
		UK	1.65 (1.57–1.75)
Rheumatic diseases [20]	Compared with RA, SLE and BS are stronger risk factors for herpes zoster (Korea; 1869)	SLE	4.29 (3.49–5.27)
		Behçet’s syndrome	4.54 (3.66–5.64)
		Infliximab	2.92 (1.72–4.89)
		Glucocorticoids	2.91 (1.72–4.89)
Heart failure [21]	Heart failure (Taiwan; 211)	All	2.07 (1.54–2.78)
		Men	2.30 (1.51–3.50)
Respiratory diseases [28]	COPD, asthma, and lung cancer are related to a high risk of contracting herpes zoster (Spain; 31,765)	COPD	1.16 (1.13–1.19)
		Asthma	1.67 (1.63–1.71)
		Lung cancer	1.68 (1.60–1.76)
UVR [29]	There is a higher risk of herpes zoster in men, especially those with a history of severe sunburn (US; 24,201)	Men	1.14 (1.02–1.29)
		Higher lifetime number of severe sunburn	1.13 (1.00–1.28)

HR, hazard ratio; CI, confidence interval; RA, rheumatoid arthritis; SLE, systemic lupus erythematosus; BS, Behçet’s syndrome; COPD, chronic obstructive pulmonary disease; UVR, ultraviolet radiation.

### 2.4. Respiratory Diseases

According to a study using German claims data, patients with asthma, coronary heart disease, chronic obstructive pulmonary disease (COPD), depression, and rheumatoid arthritis had an average of 30% increased risk of developing acute herpes zoster compared with patients without any chronic underlying conditions [30]. In a recent large population analysis using artificial intelligence, COPD, asthma, and lung cancer demonstrated a higher risk of developing herpes zoster [28] (Table 1). Regarding postherpetic neuralgia, an increased risk was only observed in patients with COPD (odd ratio (OR) 1.24 [95% CI 1.23–1.25]) and lung cancer (1.14 [1.13–1.16]). These insights underscore the intricate interplay between various chronic conditions and the susceptibility to herpes zoster, emphasizing the importance of comprehensive risk assessments and targeted preventive measures.

### 2.5. Malignancy

Profoundly compromised immune states, such as those seen in individuals with solid tumors, are strongly linked to an elevated susceptibility to herpes zoster due to a decline in VZV-specific cellular immunity [31,32]. In immunocompromised patients, particularly those with cancer, herpes zoster can lead to increased morbidity [33]. This is characterized by a prolonged phase of skin rash and an elevated risk of cutaneous dissemination, as well as involvement of the internal organs, including pneumonitis, hepatitis, and central nervous system disease [34]. Notably, the risk of developing herpes zoster is higher among patients undergoing chemotherapy [35] or immunotherapy [36] compared to individuals not currently undergoing active treatment.

### 2.6. Immunocompromised Conditions

Immune-compromised conditions, including acquired immunodeficiency syndrome (AIDS) [32], systemic lupus nephritis [37], rheumatoid arthritis [27,38], transplant recipients [39], lymphoma, leukemia, and cancer, are widely recognized as established risk factors for herpes zoster [40,41,42]. In contrast to the course of herpes zoster in immunocompetent patients, herpes zoster often recurs or is prolonged in immunocompromised patients, especially those with AIDS [43]. This heightened susceptibility underscores the need for vigilant monitoring and specialized care for immunocompromised individuals to mitigate the impact of herpes zoster and improve overall health outcomes.

### 2.7. Rheumatoid Arthritis

In a retrospective cohort study that encompassed 20,357 individuals with rheumatoid arthritis, factors contributing to the risk of herpes zoster encompassed advanced age, the use of prednisone, medications employed in the management of moderate and severe rheumatoid arthritis, as well as various comorbidities [44]. Among patients undergoing treatment with TNF-α antagonists, both etanercept (HR 0.62) and adalimumab (HR 0.53) were linked to a reduced likelihood of developing herpes zoster.

### 2.8. Inflammatory Bowel Disease

Patients with inflammatory bowel disease face a significantly elevated risk of herpes zoster [39,45]. Over an average follow-up period of 5.0 years, the likelihood of developing herpes zoster was notably higher in individuals with Crohn’s disease (adjusted HR 2.13, *p* < 0.001) and ulcerative colitis (adjusted HR 1.40, *p* < 0.001) compared to the control group. Particularly in young patients with Crohn’s disease, the incidence of herpes zoster significantly rises in those with metabolic comorbidities, such as diabetes mellitus, hypertension, or dyslipidemia.

### 2.9. Iatrogenic Risk Factors

Herpes zoster manifests in immunocompromised patients undergoing radiation therapy, bone marrow transplantation, immunosuppressive medication, or prolonged use of steroids [46]. Individuals with COPD face an elevated risk of developing herpes zoster compared to the general population. Moreover, the relative risk of herpes zoster is higher for patients with COPD using oral steroids (adjusted HR 3.00, 95% CI 2.40–3.75) [47]. Limited studies have explored the occurrence of herpes zoster during immunotherapy [36]. Immune checkpoint inhibitors can trigger herpes zoster in two ways: during the treatment of immune-related adverse events with high-dose steroids and in the context of immune reconstitution inflammatory syndrome [48]. Some Janus kinase inhibitors, particularly peficitinib, baricitinib, and tofacitinib, are linked with an increased risk of herpes zoster infection in patients with immune-mediated inflammatory diseases such as rheumatoid arthritis and inflammatory bowel disease [49].

### 2.10. Ultraviolet Radiation (UVR)

Exposure to ambient UVR was linked to an elevated risk of herpes zoster in men, but this association was not observed in women [29] (refer to Table 1). For men, a history of severe sunburn was modestly associated with an increased risk of herpes zoster, potentially due to immunosuppression resulting from excessive sun exposure.

## 3. Postherpetic Complications

**Figure 2:**
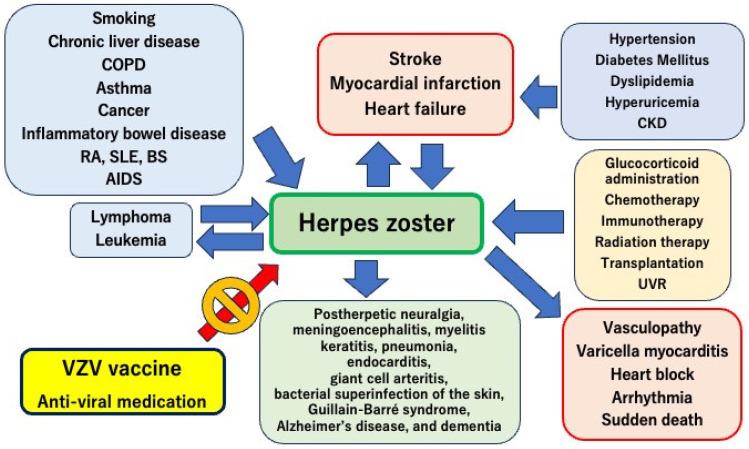
Relationship between cardiovascular disease and herpes zoster. Cardiovascular diseases, including myocardial infarction, heart failure, and stroke, are closely related to the onset and worsening of herpes zoster. Furthermore, severe complications induced by herpes zoster are important for cardiovascular diseases, such as vasculopathy, myocarditis, arrhythmia, and sudden death. Aggressive vaccination against the varicella-zoster virus is essential for the prevention and management of cardiovascular diseases. COPD, chronic obstructive pulmonary disease; RA, rheumatoid arthritis; SLE, systemic lupus erythematosus; BS, Behçet’s syndrome; AIDS, acquired immunodeficiency syndrome; VZV, varicella zoster virus; CKD, chronic kidney disease; UVR, ultraviolet radiation.

Herpes zoster is typically benign, lasting between 4 and 7 days; however, severe complications may occur. VZV infects a wide variety of cell types in the central and peripheral nervous system, thereby expanding the diversity of clinical disorders associated with the virus. Although postherpetic neuralgia is the most debilitating complication, there are several other severe postherpetic complications (Figure 2). VZV infection can cause various neurological disorders, such as meningitis, encephalitis, transverse myelitis, radiculitis, and polyneuritis [50]. However, the exact mechanisms underlying the development of postherpetic complications remain unclear.

### 3.1. Neuralgia

Postherpetic neuralgia is characterized by chronic strong pain in the area where the rash occurs. The pain is typically sharp, stabbing, or burning, and may persist for weeks to months or even years after the rash has healed. This pain can be severe and debilitating, affecting the patient’s quality of life. Postherpetic neuralgia is the leading cause of pain-related suicide among older adults [51]. Patients with advanced age and weakened immune systems, such as those with HIV or those undergoing immunosuppressive treatment, are at a higher risk. The inflammation caused by VZV during acute infection may damage or sensitize the nerves, resulting in prolonged pain signals. Early intervention for postherpetic neuralgia is often crucial to effectively manage this condition.

Furthermore, the risk of postherpetic neuralgia is higher in women and increases significantly with age and comorbidities such as diabetes mellitus, COPD, and heart failure [52]. The cumulative risk of developing herpes zoster between the ages of 50 and 90 years was found to be 31.7%. A comprehensive medical approach to improving the overall health of individuals susceptible to shingles and its complications is essential, necessitating the addressing of risk factors for postherpetic neuralgia.

### 3.2. Meningoencephalitis

Herpes zoster encephalitis induced by viral dissemination into the central nervous system is a potential complication that can lead to virus-induced cerebral angiitis [53]. The presentation is similar to most cases of viral encephalitis and includes confusion, headache, fever, and meningeal symptoms [14]. Recognizing these neurological complications is crucial for prompt diagnosis and intervention. In summary, herpes zoster can lead to encephalitis with associated cerebral angiitis, highlighting the importance of vigilance in identifying and managing these potential complications for better patient outcomes.

### 3.3. Myelitis

When VZV spreads centrally along peripheral nerves toward the spinal cord, it can enter the spinal cord at levels corresponding to the affected dorsal root ganglia and nerves, leading to myelitis [14]. If the cervical spinal cord is affected, individuals with zoster myelitis may experience weakness in all four extremities and impaired sphincter function. Herpes zoster with a sacral distribution can also result in bladder and bowel dysfunctions. Moreover, zoster myelitis with a cervical or thoracic distribution may induce diaphragmatic paralysis, which should be considered a potential cause of death in patients with VZV infection-induced cervical radiculitis or myelitis.

### 3.4. Cranial Nerve Damage

Ocular herpes zoster affects the ophthalmic branch of the trigeminal nerve, leading to significant local eye complications [15]. Moreover, the reactivation of the VZV along the seventh cranial nerve can result in the Ramsay Hunt syndrome, characterized by facial paralysis and otic zoster. Both ocular herpes zoster, which leads to significant eye complications, and Ramsay Hunt syndrome profoundly impact a patient’s quality of life. Healthcare professionals managing these conditions should prioritize early intervention whenever feasible. Increasing awareness about these illnesses is crucial as this can facilitate early treatment by ensuring that more people are informed about these conditions.

### 3.5. Keratitis

The reactivation of herpes zoster ophthalmicus has the potential to result in peripheral ulcerative keratitis. Concurrently, herpes zoster keratitis may manifest alongside other eye conditions, including blepharitis, conjunctivitis, scleritis, uveitis, and retinopathy [54]. VZV can trigger immune-mediated damage across all layers of the cornea, leading to scarring, thinning, and the development of new blood vessels (neovascularization) [55]. Ultimately, individuals with herpes zoster keratitis may experience visual impairment and, possibly, progression to blindness [56]. By recognizing these complications, early intervention and comprehensive eye care can be provided to individuals affected by herpes zoster.

### 3.6. Respiratory Complications

Primary VZV infection in adults tends to have a more severe manifestation, notably including interstitial pneumonia, compared to younger individuals. Varicella pneumonia, occurring as a complication of herpes zoster, is infrequent and is predominantly documented in immunocompromised individuals. Cases of varicella pneumonia linked to VZV infection are often associated with a high incidence of respiratory failure. Nevertheless, timely diagnosis and the prompt initiation of antiviral medications have the potential to enhance outcomes [57].

### 3.7. Subacute Endocarditis

An unusual occurrence of subacute bacterial endocarditis subsequent to herpes zoster has been documented [58]. It is important to highlight that herpes zoster has been linked to the onset of other significant infections, such as pneumonia and necrotizing fasciitis.

### 3.8. Giant Cell Arteritis

Giant cell arteritis, also recognized as temporal arteritis, is a chronic granulomatous inflammation affecting medium and large blood vessels, with the potential for severe, visual, and life-threatening consequences [59]. Although recent data indicate that herpes zoster might serve as an underlying immunological trigger for giant cell arteritis, the role of VZV in the development of giant cell arthritis remains a subject of controversy [14]. The prevalence of giant cell arteritis was notably higher among individuals with herpes zoster compared to the general population (0.340% vs. 0.143%, *p* < 0.0001) [60]. Conducting additional research to clarify the connection between herpes zoster and giant cell arteritis is anticipated to facilitate the early detection and proper management of this severe vascular disease.

### 3.9. Bacterial Superinfection of the Skin and Soft Tissue

Primarily caused by Group A Streptococcus, superinfection of the skin and soft tissue is induced by bacteria. Distinguishing herpes zoster skin rashes from bacterial epidermal and facial soft tissue infections can be challenging in clinical practice. Moreover, bacterial superinfection of herpes zoster may complicate diagnoses [61]. When the differential diagnosis is difficult, C-reactive protein and leukocyte counts should be determined, whereas parameters such as neutrophils or immature granulocytes do not add diagnostic value.

### 3.10. Guillain–Barré Syndrome

There appears to be an association between Guillain–Barré syndrome and preceding herpes zoster. Despite the availability of numerous case reports on this link, establishing a causal relationship remains challenging, given the common occurrence of both conditions among neurologists [14]. The time span between the initial zoster rash and the onset of inflammatory polyradiculitis varies widely, ranging from 2 days to several months. Clinical symptoms mirror those typically observed in Guillain–Barré syndrome, and a brief interval (less than 2 weeks) between the rash and Guillain–Barré syndrome is indicative of a poor prognosis. The treatment for this complication aligns with that employed for Guillain–Barré syndromes related to other causes.

### 3.11. Alzheimer’s Disease and Dementia

In a comprehensive population-based cohort study conducted in Korea, the presence of the herpes virus was linked to an elevated risk of dementia [62]. Conversely, the utilization of antiviral agents was associated with a reduced risk of dementia in individuals with herpes zoster. Herpes viruses, specifically herpes simplex virus type 1 and VZV, have been implicated in the development of Alzheimer’s disease. Antivirus treatment demonstrated an association with a decreased risk of dementia (adjusted HR 0.89, 95% CI 0.86–0.92), while herpes infection without antiviral medications increased the risk of dementia (adjusted HR 1.50, 95% CI 1.29–1.74) [63]. Consequently, antiviral treatment is linked to a diminished long-term risk of dementia in individuals displaying evident signs of herpes infection. Conversely, a meta-analysis comprising nine studies found no significant association between herpes zoster and the incidence of dementia or Alzheimer’s disease [64]. However, it did reveal a noteworthy association between herpes zoster opthalmicus and the incidence of dementia [64,65]. 

### 3.12. Subsequent Cancer Risk

The herpes zoster group exhibited a significantly increased risk of hematologic malignancies, including multiple myeloma (HR 1.63, 95% CI 1.37–1.95), leukemia (HR 1.20, 1.03–1.39), and lymphoma (HR 1.15, 11.02–1.30), compared with the non-herpes zoster group [66]. Conversely, the risk of liver (HR 0.87, 95% CI 0.82–0.93) and larynx (HR 0.73, 0.58–0.92) cancers was significantly decreased in the herpes zoster group compared to the non-herpes zoster group. 

The herpes zoster with the postherpetic neuralgia group demonstrated a higher HR for specific cancer risk, such as lymphoid and hematopoietic systems (95% CI 1.27–2.39, *p* < 0.001). However, no significant increase in the HR for cancers of the lips, mouth, pharynx, digestive system, and respiratory system was observed [67].

## 4. Antiviral Treatment for Herpes Zoster

The treatment of herpes zoster may be challenging and is aimed at reducing symptoms and improving patients’ quality of life. However, according to a recent retrospective cohort study, antiviral treatment did not positively affect the long-term survival of herpes zoster patients [18]. Additionally, data from Danish national registers revealed that individuals who had received treatment for herpes zoster had a 127% increased risk of stroke in the first 2 weeks, especially in the youngest age group (<40 years) [68]. Drugs used for the treatment of herpes zoster can induce coronary artery spasm. Thymidine analog rivudine is an antiviral medication used to treat herpes zoster. Its molecular structure is similar to that of 5-fluorouracil (5-FU), which has been linked to coronary spasm in the past [69]. Nevertheless, other studies have reported positive effects of antiviral medication on cardiovascular outcomes in patients with herpes zoster in the short [70,71,72,73] and long term [68]. Notably, absence of antiviral treatment is associated with a higher incidence of stroke in patients with herpes zoster [71].

## 5. Prevention of Herpes Zoster and Its Complications

The most efficient means to prevent postherpetic neuralgia is by mitigating the risk of developing herpes zoster through vaccination. Recognized as a vaccine-preventable ailment, herpes zoster vaccination is recommended for individuals aged > 50 years and has demonstrated efficacy in reducing the incidence of both herpes zoster and postherpetic neuralgia. 

Vaccines targeting varicella-zoster, including Varivax for varicella and Shingrix and Zostavax for herpes zoster, are accessible options for preventing herpes zoster [74]. The recombinant zoster vaccine (RZV) has exhibited sustained high efficacy against herpes zoster, remaining effective approximately 10 years post-vaccination, with a clinically acceptable safety profile [75]. Studies have confirmed the effectiveness of the zoster vaccine in preventing incident herpes zoster in older individuals with chronic kidney disease [76]. Recent global vaccination guidelines have recently been revised to prioritize recombinant zoster vaccines for high-risk groups, such as patients with cancer [77]. The immunogenicity of the recombinant zoster vaccine has been explored in patients with cancer undergoing chemotherapy, revealing that both humoral and cell-mediated immune responses persisted 12 months post-vaccination [78]. Compared to the general population, older adults with immune senescence and those who are immunocompromised due to disease or immunosuppressive therapy face an elevated risk of herpes zoster and its complications, which can be severe and life-threatening. Vaccination emerges as an effective strategy against herpes zoster, with studies demonstrating that herpes zoster vaccination in immunocompromised individuals can induce immune responses and offer protection from infection [79].

## 6. Recurrence Rate of Herpes Zoster

Herpes zoster was traditionally thought to occur only once in a lifetime, with recurrences mainly observed in severely immunocompromised individuals. However, there is a growing trend in the recurrence rate, which is now reported as 12.0 per 1000 person-years. In a population-based study conducted in Korea, several notable risk factors for the recurrence of herpes zoster were identified. These included old age (51–70 years) (HR 1.447, 95% CI 1.311–1.598), female gender (HR 1.476, 95% CI 1.345–1.619), and zoster-related pain lasting longer than 90 days (HR 2.293, 95% CI 1.990–2.643) [80]. Additionally, concurrent conditions such as hematologic malignancies (HR 2.864, 95% CI 1.929–4.251), autoimmune diseases (HR 1.466, 95% CI 1.252–1.715), dyslipidemia (HR 1.390, 95% CI 1.263–1.530), and hypertension (HR 1.222, 95% CI 1.107–1.350) were identified as significant risk factors for the recurrence of herpes zoster.

## 7. Herpes Virus-Induced Vasculopathy

VZV vasculopathy encompasses instances of varicella or zoster that exhibit a temporal association with stroke, particularly when the zoster occurs within the ophthalmic division of the trigeminal nerve [81]. 

### 7.1. Vasculopathy-Related Diseases

VZV vasculopathy, which has the potential to trigger stroke, giant cell arteritis, and granulomatous aortitis, arises from productive viral infection of arteries [81]. The presence of VZV within the sensory ganglia allows for transaxonal travel to cerebral arteries, where the nerve terminates in the adventitia, as well as to coronary arteries [82,83]. This process is accompanied by an increased sympathetic tone, elevated blood pressure, and altered immunological status [84]. These mechanisms can initiate inflammation and pathological vascular remodeling [85], ultimately leading to vascular failure, atherosclerosis, microvascular angina, and heart failure induced by microvascular dysfunction.

### 7.2. Vasculopathy-Related Signaling

The inflammation induced by VZV in arteries plays a role in pathological vascular remodeling. This process involves the downregulation of programmed death ligand-1, contributing to persistent vasculitis [81]. Additionally, it involves major histocompatibility complex class-I, which leads to ineffective clearance of the virus. 

### 7.3. Pathological Changes

Arteries infected with VZV exhibit a disrupted internal elastic lamina, a thickened intima composed of myofibroblasts expressing α-smooth muscle actin, and a deficiency of medial smooth muscle cells, resulting in the compromise of vessel wall integrity [86]. In the early stages of VZV vasculopathy, arteries contain a substantial number of neutrophils in the adventitia, a presence that diminishes in the later stages of VZV vasculopathy [87].

## 8. Mechanisms of Herpes Zoster-Induced Cardiovascular Diseases

Examinations of herpes zoster through pathological analyses have revealed diverse levels of ganglionic hemorrhage and inflammation [88]. Observations from reports have outlined the existence of hemorrhage, necrosis, and inflammation within the spinal ganglia affected by herpes zoster in patients [89,90]. The histological characteristics encompass mononuclear and lymphocytic inflammation, neuronal degeneration, phagocytosis of neurons by satellite cells, and vacant neuronal cell beds. This process ultimately leads to fibrous scarring of the ganglia [46].

### 8.1. Inflammation

The infection of herpes zoster initiates an inflammatory response within the body. The release of proinflammatory cytokines during herpes zoster infection triggers endovascular inflammation, leading to the rupture of atherosclerotic plaque [74]. Subsequently, thrombosis occurs, resulting in acute cardiovascular events such as acute myocardial infarction [91]. Cerebrospinal fluid obtained from virologically confirmed VZV vasculopathy patients exhibited significantly elevated levels of interleukin (IL)-8, proinflammatory cytokines (IL-6, IL-1β, TNF-α, IFN-γ, IL-2), anti-inflammatory cytokine IL-10, and matrix metalloproteinases-2 [92]. IL-8 serves as a chemoattractant for neutrophils, while IL-6 promotes macrophage differentiation, potentially contributing to inflammation and damage to the vascular wall-key features of VZV vasculopathy [81]. Therefore, the chronic inflammation induced by herpes zoster vasculopathy is linked to an elevated risk of cardiovascular diseases, including atherosclerosis.

### 8.2. Autonomic Nervous System Dysfunction

VZV infection seldom leads to orthostatic hypotension, characterized by widespread sympathetic dysfunction due to polyneuropathy [93]. In contrast, VZV infection results in dysfunction of the bladder and bowel, involving segmental dysfunction of the parasympathetic or sympathetic system due to dorsal root ganglionopathy. Recognizing autonomic dysfunction associated with VZV is crucial, particularly because individuals experiencing such symptoms may seek medical attention at gastroenterology or urology clinics. Autonomic dysfunction induced by herpes zoster may also contribute to cardiovascular complications, including arrhythmias and elevated blood pressure.

### 8.3. Vasculopathy

Herpes zoster is linked to various vasculopathies, encompassing ischemic stroke, aneurysm, cerebral and subarachnoid hemorrhage, mononuclear vision loss, arterial dissection, peripheral arterial disease, myocardial infarction, and transient ischemic stroke [81,85,94,95]. The complex relationship between herpes zoster and various vascular problems emphasizes the importance of thorough medical care and preventive measures for these patients. It is important to understand the potential impact on various vascular complications. Continued research is essential to uncover the underlying mechanisms and enhance strategies to manage these complex connections, ultimately leading to better outcomes for patients.

## 9. Herpes Zoster-Related Cardiovascular Diseases

Evidence suggesting the involvement of herpes zoster in the development of cardiovascular diseases has recently been reported [18]. Specifically, activation of the immune system during herpes zoster may cause vascular inflammation and heart failure. Understanding and managing this association is essential for the prevention of cardiovascular disease (Figure 2).

### 9.1. All Major Adverse Cardiac and Cerebrovascular Events (MACCEs)

A recent retrospective cohort study analyzing the association between herpes zoster and MACCEs showed that cumulative MACCE survival at the end of the follow-up period was 54.4% in patients with herpes zoster compared with 74.2% in the non-herpes zoster participants [18]. Although contracting herpes zoster has been shown to increase the risk of MACCEs within a year after infection, there is no relationship between the presence or absence of herpes zoster and the risk of cerebral infarction. The higher risk of MACCEs may be due to systemic unhealthy conditions, such as dehydration, insomnia, temporal hypertension, and mental stress induced by the strong pain of neuralgia. A study identified 29,054 cases of herpes zoster among 16,811,501 in-hospital cases registered from 1208 hospitals. Factors associated with a poor short-term prognosis in this group included age over 75 years (HR 2.18, 95% CI 1.55–3.05), liver cirrhosis or hepatic failure (HR 1.65, 95% CI 1.22–2.24), chronic kidney disease (HR 1.92, 95% CI 1.10–3.34), heart failure (HR 1.65, 95% CI 1.22–2.24), and old cerebrovascular events (HR 1.92, 95% CI 1.10–3.34) [19]. 

### 9.2. Stroke

Several studies and meta-analyses have demonstrated an increased risk of stroke following herpes zoster infection [18,70,71,72,83,84,91,96,97,98,99,100,101,102,103]. The stroke risk increases in the first week following herpes zoster infection and then decreases over the subsequent 6–12-month follow-up period [104]. The Taiwan National Health Research Institute records revealed a 4.5-fold increased risk of stroke if herpes zoster occurred in the ophthalmic division of the trigeminal nerve [105]. In patients aged < 40 years with herpes zoster, the risks of stroke and transient ischemic attacks were significantly higher (1.74- and 2.42-fold, respectively) [106].

In long-term analyses, the multivariable-adjusted HR for stroke was 1.38 (95% CI 1.19–1.74) in individuals 5–8 years after the herpes zoster occurrence compared with those with no history of herpes zoster [100] (Table 2). The risk of stroke associated with herpes zoster potentially varies depending on the focus and extent of the lesion and severity of pain.

### 9.3. Myocardial Infarction

Numerous clinical studies have indicated that herpes zoster raises the likelihood of experiencing myocardial infarction [70,72,83,84,91,95,96,97,98,99,107]. Over a mean follow-up duration of 4.4 years, the cumulative survival rate for AMI was 68.7% in the herpes zoster group and 90.0% in the non-herpes zoster group [18]. Herpes zoster infection was linked to a 1.35-fold increased risk of myocardial infarction within the initial 30 days post-infection [74]. Factors such as history of prior myocardial infarction, male gender, age over 50 years, a history of heart failure, peripheral vascular disease, HIV infection, prior cerebrovascular accident, and renal disease heightened the odds of experiencing a myocardial infarction within 30 days after herpes zoster infection.

### 9.4. Coronary Heart Disease

As shown in Table 2, long-term analyses comparing individuals with no history of herpes zoster revealed that the multivariable-adjusted HR for coronary heart disease was 1.25 (95% CI 1.07–1.46) among individuals 9–12 years after the herpes zoster occurrence [100]. Infection-associated systemic inflammation and increased heart rate are known to increase the metabolic demands on systemic organs and tissues and decrease coronary perfusion. These conditions are crucial for the development of coronary artery disease.

### 9.5. Myocarditis

Direct damage to the myocardium caused by the virus and immunological abnormalities following infection are deemed to cause viral myocarditis. Varicella myocarditis is a serious form of VZV that causes cardiac complications [108,109,110]. Although myocarditis caused by VZV is rare, it is an important complication associated with high mortality.

### 9.6. Heart Failure

In a retrospective case–control study based on the National Health Insurance System claims database of Korea, of 20,311 patients with herpes zoster, severe herpes zoster requiring hospitalization independently increased the risk of subsequent heart failure (HR 2.034, 95% CI 1.615–2.562) [22]. Importantly, heart failure was also a major risk factor for herpes hospitalization (HR 1.485, 95% CI 1.041–2.117).

### 9.7. Arrhythmia

VZV has been documented to necessitate permanent pacemaker placement due to complete heart block [111,112,113] and lead to second-degree atrioventricular block [114]. The development of a complete heart block is believed to result directly from the reactivation of the virus within the cardiac sympathetic/parasympathetic ganglia [111]. Comprehending and consistently anticipating these cardiac complications is essential for timely intervention and the effective management of patients with shingles. Additional research is necessary to delve into the mechanisms underlying VZV-related cardiac issues and optimize prevention strategies for this specific group of patients.

## 10. Conclusions

Herpes zoster, a condition that significantly diminishes quality of life, is preventable through vaccination. Recent studies have unveiled associations with prolonged pain, severe cardiovascular disease, and stroke. Therefore, vaccination against VZV is strongly recommended, especially for individuals over the age of 50. In conclusion, recognizing herpes zoster as a substantial concern in cardiovascular disease management, a more proactive vaccination approach is crucial to mitigate its impact and enhance overall health outcomes.

## Figures and Tables

**Table 2:** Cardiovascular diseases and history of herpes zoster.

Disease [Reference]	Results (Country, Incident)	Years Since Herpes Zoster	HR (95% CI)
Stroke [100]	Higher long-term risk of stroke (US, 3603)	1–4 years	1.05 (0.88–1.25)
		5–8 years	1.38 (1.10–1.74)
		9–12 years	1.28 (1.03–1.59)
		≥13 years	1.19 (0.90–1.56)
Coronary heart disease [100]	Higher long-term risk of coronary heart disease (US, 8620)	1–4 years	1.13 (1.01–1.27)
		5–8 years	1.16 (1.02–1.32)
		9–12 years	1.25 (1.07–1.46)
		≥13 years	1.00 (0.83–1.21)
Cardiovascular disease [22]	Severe herpes zoster is associated with cardiovascular disease (Korea, 20311)	Myocardial infarction	1.83 (1.35–2.48)
		Ischemic stroke	1.52 (1.21–1.92)
		Heart failure	2.03 (1.62–2.56)

HR, hazard ratio.
